# Psychometric properties of AMAS and math anxiety prevalence among Chinese and Russian schoolchildren: a comparative study

**DOI:** 10.3389/fpsyg.2024.1485753

**Published:** 2024-12-20

**Authors:** Du Linna, Wang Xinghua, Yu Haiying, Anna Pavlova, Victoria Ismatullina, Artem Malykh, Pavel Kolyasnikov, Sergey Malykh

**Affiliations:** ^1^School of Education and Science, Mudanjiang Normal University, Mudanjiang, China; ^2^Oriental Language Institute, Mudanjiang Normal University, Mudanjiang, China; ^3^Center for Interdisciplinary Research in the Educational Sciences, Russian Academy of Education, Moscow, Russia; ^4^Behavior Genetics Laboratory, Psychological Institute of Russian Academy of Education, Moscow, Russia; ^5^Center of Population Research, Ural Institute of Humanities, Ural Federal University, Ekaterinburg, Russia

**Keywords:** AMAS, math anxiety, schoolchildren, psychometric properties, cross-cultural comparison

## Abstract

The purpose of this study was to compare the prevalence of math anxiety in Russian and Chinese schoolchildren across genders and ages. The Abbreviated Math Anxiety Scale (AMAS) was used as a measurement tool for assessing math anxiety. The factor structure of the AMAS and item invariance between Russian and Chinese schoolchildren were also examined. A total of 4,292 Russian (54% girls, *M* = 13.7, SD = 1.21) and 3,410 Chinese (48% girls, *M* = 12.7, SD = 1.21, Me = 13.0) schoolchildren participated in the study. The bi-factor model of the AMAS fits provided the best fit for the data in both countries. AMAS items demonstrated invariance between the two groups. Overall, Russian schoolchildren demonstrated higher math anxiety across all ages and math anxiety subscales, except at ages 14–15, where Chinese schoolchildren reported higher learning-related math anxiety. Among Chinese schoolchildren, both learning and evaluation math anxiety increased with age. Conversely, for Russian schoolchildren, math evaluation anxiety increased, while learning math anxiety decreased with age. Gender differences were observed in both countries, with the onset of gender-related differences appearing earlier in Chinese schoolchildren.

## 1 Introduction

Math anxiety can be defined as an intensive negative emotional experience associated with math-related tasks (for example, manipulation of numbers) (Richardson and Suinn, 1972). Academic literature dedicated to math anxiety has increased rapidly in recent years (Ersozlu and Karakus, 2019; Sagarduy et al., 2024). According to bibliometric research (Radević and Milovanović, 2024), the majority of studies are dedicated to cognitive correlates of math anxiety, psychological effects, and educational contexts.

Math anxiety may have a negative impact on math achievements (Carey et al., 2016; Schmader et al., 2008; Park et al., 2014; Luo et al., 2014; Barroso et al., 2020; Buzzai et al., 2020; Zhang et al., 2019) and, as a phenomenon, exists across different cultural contexts (Cipora et al., 2015; Brown et al., 2020; Martín-Puga et al., 2022; Coronado-Hijón, 2017).

There are several cross-cultural comparative studies on math anxiety, such as a comparison between Russia and the UK (Rudenko et al., 2013), Confucian and European countries (Morony et al., 2013), the US and Colombia (Brown et al., 2020), Finland, the US, and Korea (Fan et al., 2019), and Germany and Brazil (Wood et al., 2012). The studies provide evidence for a strong cultural influence on math anxiety. For example, students in Confucian countries report higher math anxiety in comparison with European countries (Morony et al., 2013). In the present study, we expect to see differences in math anxiety scores between Chinese and Russian schoolchildren due to cultural and educational system differences.

One of the salient results from studies of math anxiety is that girls are more prone to exhibit high math anxiety in comparison to boys (Primi et al., 2014; Sadiković et al., 2018). Several studies (Carey et al., 2017; Schmitz et al., 2022) highlight an increase in math anxiety with age. We also expect to trace those patterns in the current study.

Abbreviated Math Anxiety Scale (AMAS), developed from Mathematics Anxiety Rating Scale (MARS) by Hopko et al. (2003), was validated for different cultural contexts, including Arabic (Megreya et al., 2023), Serbian (Milovanović and Branovački, 2021), Turkish (Kul et al., 2024), Polish (Cipora et al., 2015), Spanish (Brown et al., 2020), Russian (Marakshina et al., 2023, 2024), Italian (Caviola et al., 2017), German (Schillinger et al., 2018), and Chinese (Hongxia et al., 2022). There is some evidence for age and gender invariance of AMAS (Primi et al., 2014; Cohen and Limbers, 2022; Hongxia et al., 2022; Martín-Puga et al., 2022; Marakshina et al., 2024), as well as evidence for culture invariance in Italian and English contexts (Primi et al., 2020). Among well-established findings from AMAS studies is a higher level of math anxiety for girls (Marakshina et al., 2023, 2024; Sadiković et al., 2018; Schmitz et al., 2022), especially in older children (Primi et al., 2020). Results regarding age differences are less consistent: while some studies report an increase in math anxiety with age (Schmitz et al., 2022), others highlight an opposite tendency (Primi et al., 2014).

The purpose of the current study is to compare AMAS math anxiety scores for Chinese and Russian schoolchildren. Additionally, factor structure and invariance for Russian and Chinese cultural contexts of AMAS are measured to justify the applicability of AMAS for cross-cultural comparison.

### 1.1 Sample

The total sample consisted of 7,702 participants (52% girls, *M* = 13.2, SD = 1.36, Me = 13.0). The Russian sample comprised 4,292 participants (54% girls, *M* = 13.7, SD = 1.21, Me = 14.0). The Chinese sample comprised 3,410 participants (48% girls, *M* = 12.7, SD = 1.21, Me = 13.0). The socio-demographic data and age distribution of samples are shown in Supplementary Table 1 and Supplementary Figure 1.

### 1.2 Instruments

The Abbreviated Math Anxiety Scale (AMAS) is a 9-item questionnaire developed by Hopko et al. (2003) based on the Mathematics Anxiety Rating Scale (MARS). It comprises two subscales: one addressing learning math anxiety (LMA, consisting of five items, e.g., “I feel anxious listening to a lecture in math class”) and the other focusing on math evaluation anxiety (MEA, consisting of four items, e.g., “I feel anxious taking an examination in a math course”). Respondents are required to assess their anxiety levels for each situation described in the statements on a Likert scale ranging from 1 (low anxiety) to 5 (high anxiety). The Russian version of the AMAS has demonstrated strong psychometric properties for middle and high schoolchildren (Marakshina et al., 2023) and for high schoolchildren (Marakshina et al., 2024). Similarly, the Chinese version of the AMAS has been validated for primary and middle schoolchildren (Hongxia et al., 2022).

### 1.3 Statistical analysis

Statistical analysis was performed in Python (v 3.9) (Van Rossum and Drake, 2009) and JASP software (JASP Team, 2024). Basic descriptive statistics were used to assess differences in math anxiety in Russian and Chinese samples. Confirmatory Factor Analysis (CFA) was performed to assess the factor structure of AMAS for the two samples. The DWLS (Diagonal Weighted Least Squares) estimator was used. One-factor, two-factor, second-order (two factors), and bi-factor (two factors) CFA models were compared. TLI, CFI, RMSEA, and SRMR metrics were used to assess model fit. Structural Equation Modeling (SEM) analysis was performed to measure invariance, and the Cronbach Alpha coefficient was used to assess the internal consistency of AMAS and subscales for two samples.

## 2 Results

### 2.1 Factor structure

For all samples (the joint, Russian, and Chinese), the bi-factor model with two factors fit the data best (see Table 1). Two-factor and second-order models demonstrate acceptable fit statistics in all samples. Factor loadings of the bi-factor model for two samples are presented in Supplementary Tables 3, 4 and Supplementary Figure 2 depict the bi-factor model.

**Table 1:** Fit indices for 1 factor, 2-factor, second-order and bi-factor models in the joint, Russian, and Chinese samples.

Sample		1 factor	2 factors	Second-order: 2 factors	Bifactor: 2 factors
Join	*χ* ^2^	–	–	–	–
Russian		1,549.290 (20,910.727)	8,045.572 (20,910.727)	400.184 (20,910.727)	42.226 (20,910.727)
Chinese		220.871 (13,730.438)	91.695 (13,730.438)	91.695 (13,730.438)	20.865 (13,730.438)
Join	*p*-value				
Russian		<0.001	<0.001	<0.001	0.010
Chinese		<0.001	<0.001	<0.001	0.141
Join	TLI	0.92	0.98	0.98	0.99
Russian		0.90	0.98	0.98	0.99
Chinese		0.98	0.99	0.99	0.99
Join	CFI	0.94	0.99	0.99	0.99
Russian		0.93	0.98	0.98	0.99
Chinese		0.99	0.99	0.99	1.00
Join	RMSEA CI 90%	0.096 [0.093; 0.1]	0.051 [0.047; 0.054]	0.052 [0.048, 0.056]	0.023 [0.019; 0.028]
Russian		0.116 [0.111; 0.121]	0.059 [0.054; 0.064]	0.060 [0.055; 0.065]	0.014 [0.005; 0.022]
Chinese		0.046 [0.040; 0.052]	0.027 [0.021; 0.033]	0.028 [0.022; 0.034]	0.011 [0; 0.021]
Join	SRMR	0.11	0.049	0.049	0.021
Russian		0.125	0.054	0.054	0.014
Chinese		0.055	0.033	0.033	0.016
Join	Omega coeff	–	–	–	0.98
Russian		–	–	–	0.98
Chinese		–	–	–	0.97

### 2.2 Invariance across countries

Structural equation modeling was used to assess whether AMAS works equally for two samples (e.g., whether it is reasonable to compare their scores). During the first stage, configural invariance was tested (i.e., model parameters were estimated for both countries). Secondly, metric invariance was tested (whether factor loadings are the same in Russian and Chinese schoolchildren). Results are presented in Table 2. Model 1 shows configural invariance with an acceptable fit to the data (CFI = 0.970, RMSEA = 0.069). Model 2 demonstrates metric invariance and fits the data (CFI = 0.965, RMSEA = 0.069). Thus, the data demonstrate an invariance across countries. This means that AMAS is an instrument that can be used to measure cross-cultural differences between Chinese and Russian schoolchildren.

**Table 2:** Model fit indices for measurement invariance across countries.

	AIC	BIC	CFI	RMSEA	Baseline test			Difference test				
					*χ* ^2^	df	*p*	ΔCFI	ΔRMSEA	Δ*χ*^2^	Δdf	*p*
Model 1	156,528.531	156,930.791	0.970	0.069 [0.065; 0.073]	945.989	50	<0.001					
Model 2	156,660.693	157,007.468	0.965	0.069 [0.065; 0.073]	1,094.150	58	<0.001	0.005	0.000	148.161	8	<0.001

### 2.3 Internal consistency

The total AMAS scale and both subscales demonstrate acceptable Cronbach Alpha in both samples (see Table 3). There are no differences between Russian and Chinese samples for the total AMAS scale, but subscales, especially the LMA subscale, demonstrate slightly higher internal consistency in the Russian sample.

**Table 3:** Cronbach alpha in the joint, Russian and Chinese samples.

	Joint	Russian	Chinese
AMAS	0.81	0.81	0.81
MEA	0.67	0.67	0.65
LMA	0.70	0.72	0.66

### 2.4 Descriptive statistics

Descriptive statistics for total AMAS and two subscales with frequency histograms across the sample are presented in Supplementary Table 4 and Supplementary Figure 3. It can be seen that regardless of country, the LMA score distribution is skewed to the low values, while the MEA score distribution is closer to normal distribution. MEA and LMA show moderate Pearson correlations for all samples: *r* = 0.54, *p* < 0.001 for the joint sample, *r* = 0.52 for the Russian sample, and *r* = 0.66 for the Chinese sample.

Table 4 represents the difference in scores between Russian and Chinese schoolchildren. Overall, Russian schoolchildren show significantly higher scores for AMAS and both subscales than Chinese schoolchildren (see Supplementary Figure 4).

**Table 4:** Comparison of AMAS, LMA, and MEA mean scores between Russian and Chinese schoolchildren.

	Russian	Chinese	Difference	*T*-statistic	*p*-value
AMAS	10.24	6.30	3.94	−27.4	<0.001**
LMA	2.88	2.30	0.58	−7.55	<0.001**
MEA	7.36	4.00	3.36	−39.0	<0.001**

Supplementary Table 5 represents the difference in AMAS, LMA, and MEA scores between two samples for different age groups: 10–11-year-olds, 12–13-year-olds, and 14–15-year-olds. The same pattern is observed: Chinese schoolchildren demonstrate lower math anxiety in all three age groups than Russian schoolchildren. There is an exception for the LMA subscale for the oldest age group, where Chinese schoolchildren show a higher mean score (see Supplementary Figure 5). However, the effect size is relatively small, and statistical significance is subthreshold (*p* = 0.014).

### 2.5 Age differences

Supplementary Tables 6, 7 present age differences in AMAS scores for the two samples. Notably, no significant differences in total AMAS scores are observed across age groups in the Russian sample. However, when LMA and MEA scores are analyzed separately, opposite trends emerge: LMA scores decrease between the ages of 12–13 and 14–15, while MEA scores increase with age, particularly between the ages of 10–11 and 12–13. In contrast, in the Chinese sample, both MEA and LMA scores, as well as total AMAS scores, increase significantly with age.

Due to these opposing trends in LMA scores between the Russian and Chinese samples, Chinese schoolchildren in the 14–15-year-old cohort surpass their Russian peers in mean LMA scores. Supplementary Figure 6 illustrates the age differences in AMAS, LMA, and MEA scores between Russian and Chinese schoolchildren.

### 2.6 Gender differences

Gender differences in the two samples are presented in Supplementary Table 8 and Supplementary Figure 7. In both countries, girls demonstrate higher scores for AMAS and subscales. For LMA scales, the difference between boys and girls is not significant in the samples of both countries. However, the difference becomes significant in a joint sample.

### 2.7 Differences in age-gender interaction

Supplementary Tables 9, 10 and Supplementary Figure 8 provide information on the interaction between gender and age in the two samples. It can be seen that the youngest Russian schoolchildren (ages 10–11 years) demonstrate no significant differences in mean total and subscale scores. At age 10, Russian girls have slightly lower scores than boys on the AMAS, particularly on the MEA subscale (as indicated by intersecting confidence intervals). However, this gender advantage reverses at age 11.

For AMAS and MEA, a significant gender difference in Russian schoolchildren can be seen at age 12, and then on, its magnitude remains approximately the same. For LMA, no significant gender differences were observed across all ages. On the contrary, in Chinese schoolchildren, gender differences are significant from the beginning (10–11-year-olds). Interestingly, for LMA scores in 10–11-year-olds, there is a significant gender difference (girls score higher), but it disappears in schoolchildren of older ages. Overall, gender differences in AMAS and MEA scores are more significant in the Russian sample.

## 3 Discussion

The study aimed to investigate differences in math anxiety scores between Russian and Chinese schoolchildren aged 10–15 years. Gender and age differences were estimated, as well as differences in gender-age interaction. The abbreviated Math Anxiety Scale was used as a measuring tool.

The bi-factor model fits the data best for both Russian and Chinese samples, and it has the lowest RMSEA, lowest SRMR, and highest TLI among all the models tested. A number of studies (Sadiković et al., 2018; Cohen and Limbers, 2022; Marakshina et al., 2024) report the best fit of the bi-factor model as well. Some studies, however, report two-factor (Vahedi and Farrokhi, 2011) and second-factor (Marakshina et al., 2023) models that fit the data best. One of the advantages of a bifactor model is that it simultaneously captures the general factor and separate factors for subscales (see Rodriguez et al., 2016, for review). In the case of AMAS, the bifactor model shows that it makes sense to calculate total math anxiety scores and scores for LMA and MEA separately.

It was also shown that there is an invariance across countries (i.e., AMAS measures the same construct in Russian and Chinese schoolchildren), which is consistent with previous studies on culture invariance (Primi et al., 2020). In both countries, AMAS demonstrates high internal consistency. However, LMA shows a slightly lower internal consistency for Chinese schoolchildren than the Russian subsample. It may be because the LMA subscale does not capture the specificity of math learning situations in China well.

Schoolchildren from both countries demonstrate a similar pattern: low level of learning math anxiety and moderate level of math evaluation anxiety. This pattern is consistent with other studies (Marakshina et al., 2024, 2023; Cipora et al., 2015; Carey et al., 2016). Overall, Russian schoolchildren show higher math anxiety in comparison with their Chinese peers. This tendency persists if comparison is made within age groups with one exception: 14–15-year-old Chinese children show higher levels of learning math anxiety in comparison with 14–15-year-old Russian children. The analysis of changes in math anxiety levels as a function of age reveals that in Russia, learning math anxiety decreases with age, while math evaluation anxiety increases, whereas in China, both learning and evaluation anxiety increase as children grow older.

The differing trajectories of learning math anxiety dynamics in Russia and China explain the atypical pattern observed at ages 14–15.

Lanfaloni et al. (2023) demonstrate that learning math anxiety and math evaluation anxiety, despite being correlated, can exist independently and play different roles in math achievement. The difference in the dynamic of math evaluation anxiety and learning math anxiety observed in the Russian sample is partly consistent with a finding that secondary schoolchildren show higher math evaluation anxiety and similar learning math anxiety to primary schoolchildren (Carey et al., 2017). However, some studies show that overall math anxiety increases with age, which is more consistent with our findings for the Chinese sample (Schmitz et al., 2022). Further studies are necessary to understand the origins of different dynamics of learning math anxiety in China and Russia.

In both countries, girls show significantly higher levels of math anxiety in comparison with boys on the entire scale and MEA subscale, while for LMA, no significant gender differences are observed. Notably, country differences are more pronounced than gender differences. In previous studies on Chinese (Xie et al., 2019), Russian (Marakshina et al., 2023, 2024), and other (Primi et al., 2014; Sadiković et al., 2018; Schmitz et al., 2022) populations, gender differences in math anxiety were also highlighted.

While investigating the changes in math anxiety with age for boys and girls separately (gender-age interaction), some differences between Russian and Chinese schoolchildren were observed. In Russia, there are no significant gender differences in the youngest age group, which is consistent with studies that demonstrate the absence of gender differences in primary schoolchildren (Primi et al., 2020). It is worth noting that at age 10 years, Russian girls demonstrate even lower levels of overall math anxiety and evaluation math anxiety, although the difference was not statistically significant. At age 12 years, boys demonstrate a low level of math anxiety, and this pattern is maintained in older ages. In China, that pattern was established earlier: girls score higher than boys in all ages observed in the current study.

The gender advantage reversal between 10 and 11-year-olds in Russian schoolchildren may be associated with a transition from primary to secondary school. However, further investigations are needed to understand the mechanism of such a rapid change. In general, differences between Russian and Chinese schoolchildren may be attributed to extremely high academic pressure on Chinese schoolchildren (Sun et al., 2012; Li and Li, 2010). Gender stereotypes also may play a role (Tomasetto, 2019).

Some limitations have to be acknowledged. First, a study was cross-sectional in design, whereas a longitudinal sample is more applicable for tracing trajectories of math anxiety development with age. Second, despite similar oldest and youngest ages of schoolchildren, Russian and Chinese samples differ in terms of age structure. In particular, 10–11-year-old children are underrepresented in the Russian sample, whereas 14–15-year-old children are underrepresented in the Chinese sample.

In future studies, it is important to replicate the analysis in age-balanced samples and enhance the age range to cover all years of schooling.

## Data Availability

The raw data supporting the conclusions of this article will be made available by the authors without undue reservation.
