# Supplementary material for: Psychometric properties of AMAS and math anxiety prevalence among Chinese and Russian schoolchildren: a comparative study
Source: Front Psychol. 2024 Dec 20;15:1485753. doi: 10.3389/fpsyg.2024.1485753 (PMC11697699; doi:10.3389/fpsyg.2024.1485753)
Supplement: Supplementary file 1 [file Data_Sheet_1.pdf]

Supplementary Material

Table 1. The description of the joint, Russian and Chinese samples

Sample	N	Males	Females	Mean Age +- SD	Median Age	Min-max age
Joint	7702	3737 (48%)	3965 (52%)	13.2 +- 1.36	13.0	10-15
Russian	4292	1994 (46%)	2298 (54%)	13.7+-1.21	14.0	10-15
Chinese	3410	1743 (51%)	1667 (48%)	12.7 +- 1.21	13.0	10-15

Joint	Russian	Chinese

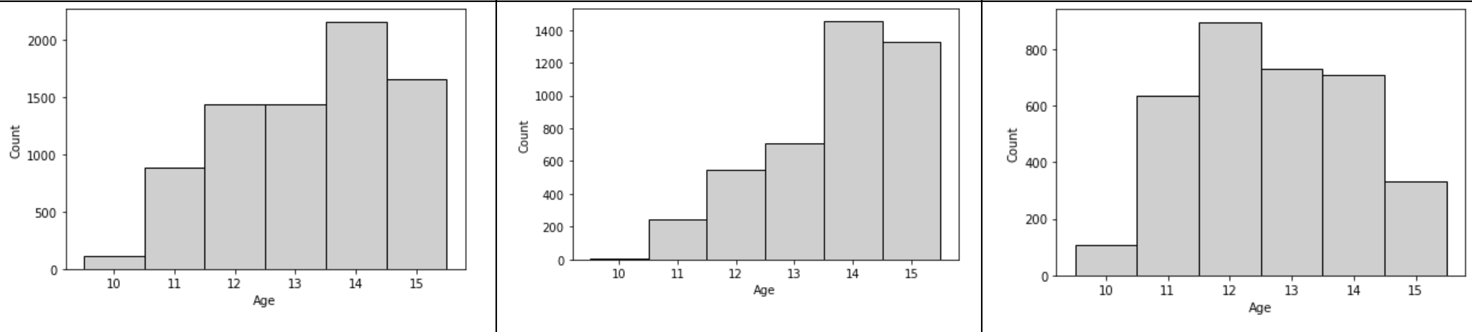

Figure 1. The age distribution of the joint, Russian and Chinese samples

Table 2. Factor loadings in bi-factor model for Russian sample

	Learning math anxiety	Math evaluation anxiety	General math anxiety
AMAS1	0.236		0.476
AMAS3	0.216		0.797
AMAS6	0.527		0.604
AMAS7	0.466		0.546
AMAS9	0.265		0.734
AMAS2		0.659	0.696
AMAS4		0.976	0.540
AMAS8		0.897	0.657
AMAS5		0.396	0.799

Table 3. Factor loadings in bi-factor model for Chinese sample

	Learning math anxiety	Math evaluation anxiety	General math anxiety
AMAS1	0.174		0.432
AMAS3	0.287		0.417
AMAS6	0.194		0.409

AMAS7	0.312		0.292
AMAS9	0.276		0.366
AMAS2		0.407	0.613
AMAS4		0.307	0.635
AMAS8		0.147	0.703
AMAS5		-0.084	0.630

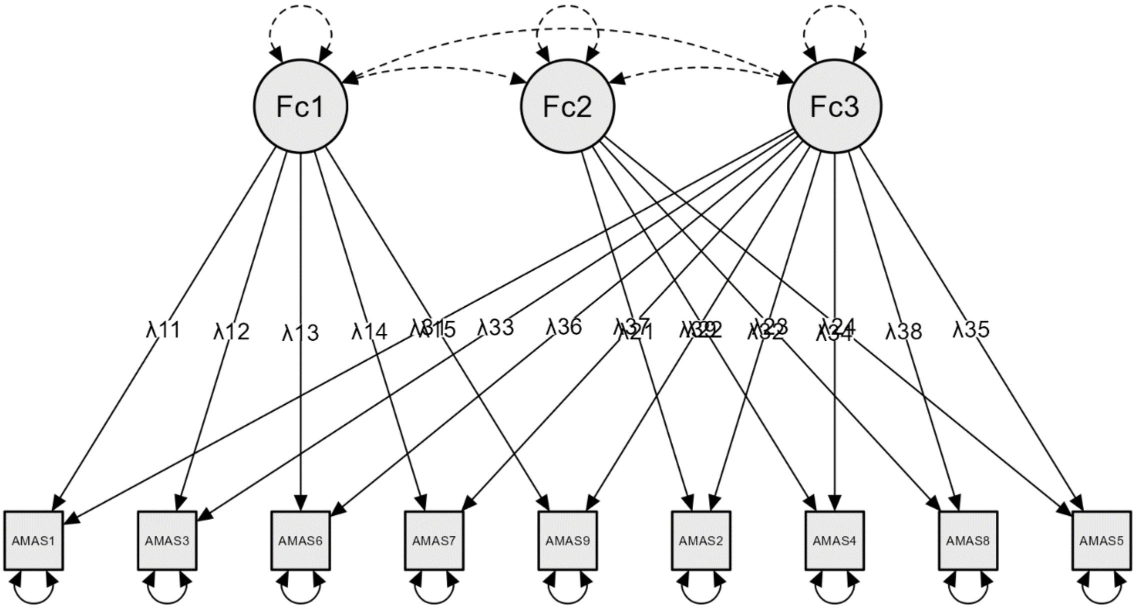

Figure 2. Model plot for factor structure of AMAS

Table 4. Descriptive statistics for AMAS, LMA, MEA across the samples

	Joint	Russian	Chinese
AMAS total			
Mean +-SD	8.47 +- 6.53	10.24 +- 7.08	6.30 +- 5.0
Median	7.0	9.0	5.0
Range	0-36	0-36	0-36
LMA			
Mean +-SD	2.62 +- 3.34	2.88 + 3.84	2.30 +- 2.56
Median	1.0	1.0	2.0
Range	0-20	0-20	0-20
MEA			
Mean +-SD	5.85 +- 4.09	7.36+4.28	4.0 +-2.9
Median	5.0	7.0	4.0
Range	0-16	0-16	0-16

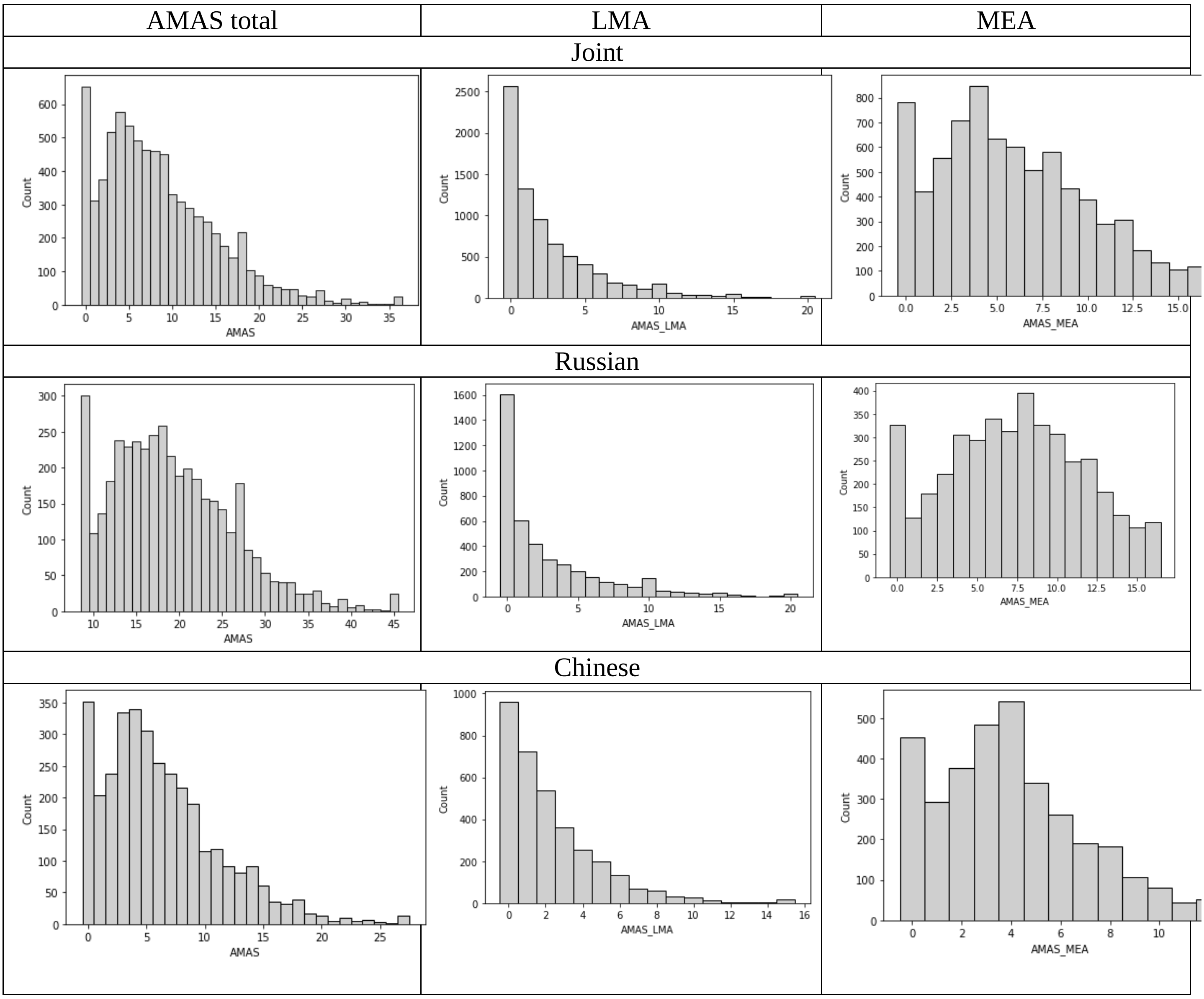

Figure 3. Score distribution for AMAS, LMA, MEA across the samples

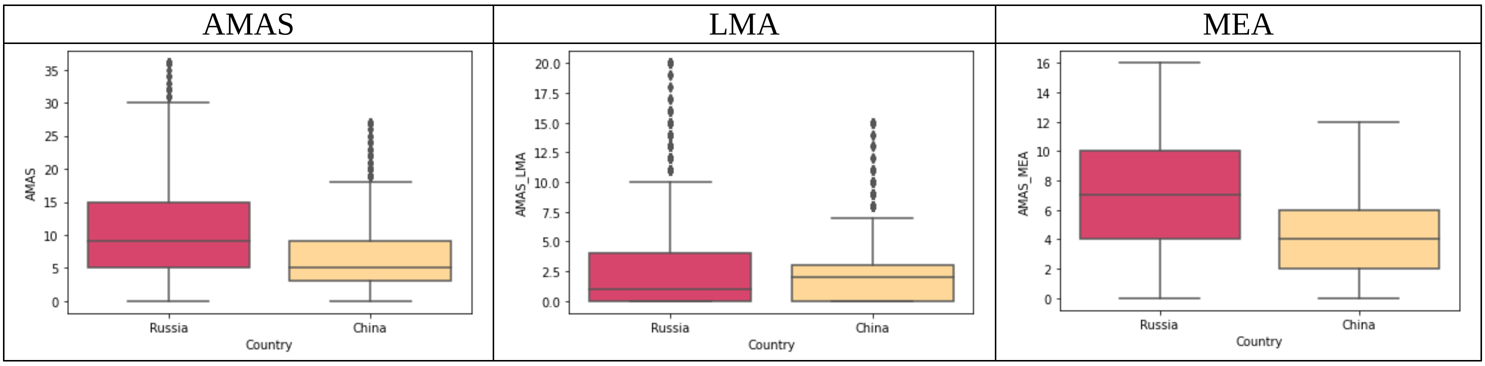

Figure 4. Boxplots of AMAS, LMA and MEA scores for Russian and Chinese schoolchildren

Table 5 Comparison of AMAS, LMA and MEA mean scores between Russian and Chinese schoolchildren in different age groups

	Russian	Chinese	Difference	T-statistic	p-value
10-11 years					
AMAS	9.79	5.54	4.25	10.76	<0.001**
LMA	3.54	1.92	1.62	8.02	<0.001**
MEA	6.25	3.62	2.63	9.70	<0.001**
12-13 years					
AMAS	10.30	5.87	4.43	19.79	< 0.001**
LMA	3.08	1.99	1.09	9.33	< 0.001**
MEA	7.21	3.87	3.34	24.5	< 0.001**
14-15 years					
AMAS	10.26	7.52	2.74	11.34	< 0.001**
LMA	2.73	3.04	-0.31	-2.44	0.014*
MEA	7.53	4.47	3.06	21.34	<0.001**

Note:

AMAS	LMA	MEA

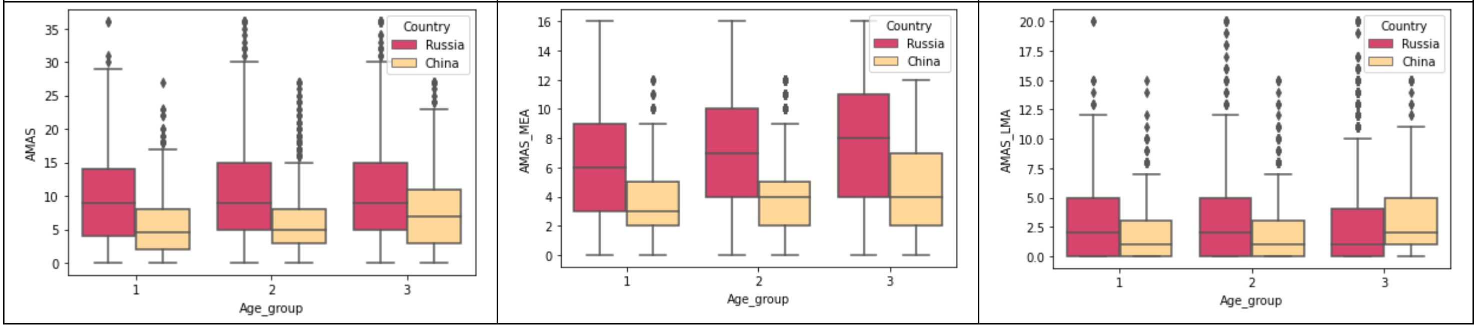

Figure 5. Boxplots of AMAS, LMA and MEA scores for Russian and Chinese schoolchildren in different ages. Note: 1 – 10-11 years old, 2 – 12-13 years old, 3 – 14-15 years old

Table 6. Comparison of AMAS, LMA and MEA mean scores between different age groups in Russian schoolchildren

	Mean younger group	Mean older group	Difference	T-statistic	p-value
AMAS					
10-11 and 12-13	9.79	10.30	-0.51	-1.01	0.31
12-13 and 14-15	10.30	10.26	0.04	0.15	0.87
10-11 and 14-15	9.79	10.26	-0.47	-1.015	0.31
LMA					
10-11 and 12-13	3.54	3.08	0.46	1.68	0.09
12-13 and 14-15	3.08	2.73	0.35	2.70	0.006**
10-11 and 14-15	3.54	2.73	0.81	3.20	0.001**
MEA					
10-11 and 12-13	6.25	7.21	-0.96	-3.17	0.002**
12-13 and 14-15	7.21	7.53	-0.32	-2.17	0.03*
10-11 and 14-15	6.25	7.53	-1.28	-4.58	< 0.001**

Table 7. Comparison of AMAS, LMA and MEA mean scores between different age groups in Chinese schoolchildren

	Mean younger group	Mean older group	Difference	T-statistic	p-value
AMAS					
10-11 and 12-13	5.54	5.87	-0.24	-1.60	0.111
12-13 and 14-15	5.87	7.52	-1.67	-8.24	<0.001**
10-11 and 14-15	5.54	7.52	-1.98	-8.01	< 0.001**
LMA					
10-11 and 12-13	1.92	1.99	-0.07	-0.73	0.46
12-13 and 14-15	1.99	3.04	-1.05	-10.25	< 0.001**
10-11 and 14-15	1.92	3.04	-1.12	-8.73	< 0.001**
MEA					
10-11 and 12-13	3.62	3.87	-0.25	-2.03	0.043*
12-13 and 14-15	3.87	4.47	-0.60	-5.10	<0.001**
10-11 and 14-15	3.62	4.47	-0.85	-6.03	<0.001**

AMAS	LMA	MEA

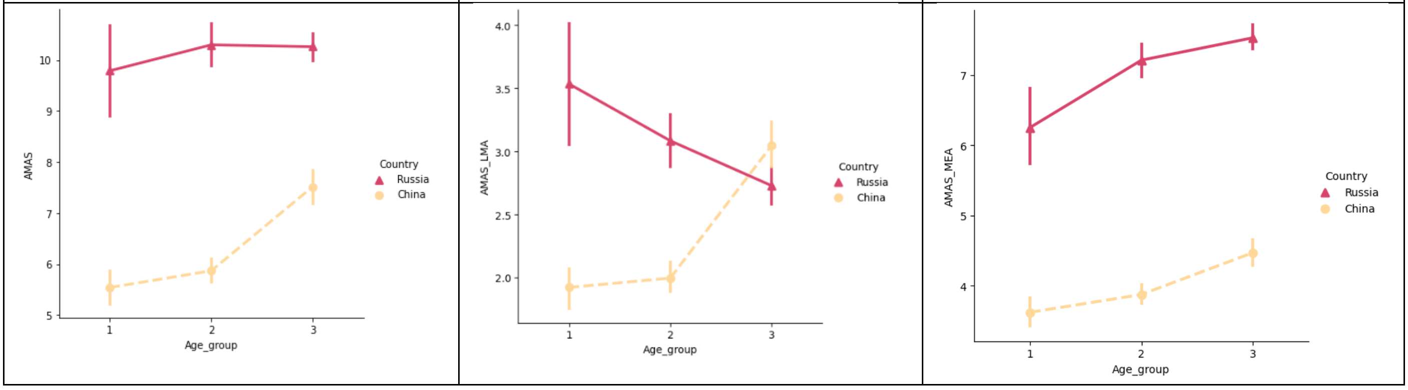

Figure 6. The change of AMAS, LMA, MEA scores as a function of age for Russian and Chinese schoolchildren. Note: 1 – 10-11 years old, 2 – 12-13 years old, 3 – 14-15 years old

Table 8. Gender differences in AMAS, LMA, MEA mean scores in the joint, Russian and Chinese samples

	Males	Females	Difference	T-statistic	p-value
Joint					
AMAS	7.56	9.32	-1.76	-11.8	<0.001**
MEA	5.06	6.60	-1.54	-16.6	<0.001**
LMA	2.51	2.72	-0.21	-2.82	0.004**
Russian					
AMAS	9.13	11.2	-2.07	-9.54	<0.001**
MEA	6.35	8.23	-1.88	-14.5	<0.001**
LMA	2.78	2.97	-0.19	-1.6	0.109
Chinese					
AMAS	5.83	6.79	-0.96	-5.57	<0.001**
MEA	3.63	4.39	-0.67	-7.71	<0.001**
LMA	2.21	2.39	-0.18	-2.11	0.034

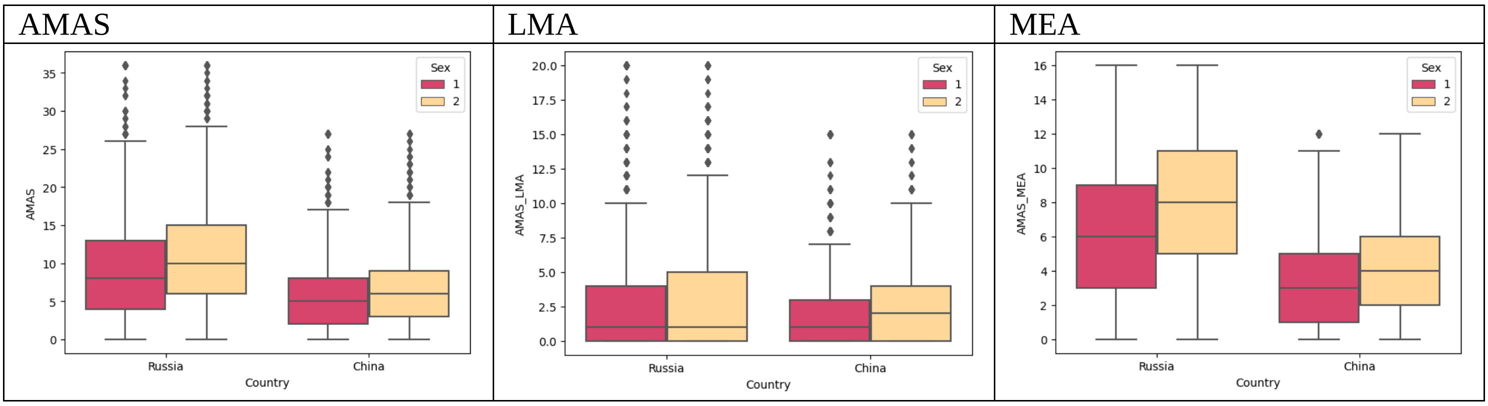

Figure 7. Boxplots of AMAS, LMA and MEA scores for Russian and Chinese schoolchildren in males and females. Note: 1 – males, 2 – females

Table 9. Gender differences in AMAS, LMA, MEA mean scores in different ages in Russian schoolchildren

	Males	Females	Difference	T-statistic	p-value
10-11 years old					
N	117	133	-	-	-
AMAS	9.05	10.44	-1.39	-1.48	0.140
MEA	5.51	6.90	-1.39	-2.59	0.010*
LMA	3.54	3.53	0.01	0.009	0.99
12-13 years old					
N	595	628	-	-	-
AMAS	9.17	11.36	-2.19	-5.35	< 0.001**
MEA	6.20	8.17	-1.97	-8.04	< 0.001**
LMA	2.97	3.19	-0.22	-0.97	0.32
14-15 years old					
N	1224	1490	-	-	-
AMAS	9.11	11.20	-2.09	-7.84	< 0.001**
MEA	6.50	8.38	-1.88	-11.8	<0.001**
LMA	2.61	2.82	-0.21	-1.46	0.14

Table 10. Gender differences in AMAS, LMA, MEA mean scores in different ages in Chinese schoolchildren

	Males	Females	Difference	T-statistic	p-value
10-11 years old					
N	369	375	-	-	-
AMAS	4.99	6.08	-1.09	-3.27	< 0.001**
MEA	3.24	3.99	-0.75	-3.74	< 0.001**
LMA	1.75	2.09	-0.34	-2.04	0.041*
12-13 years old					
N	832	795	-	-	-
AMAS	5.41	6.35	-0.94	-4.04	< 0.001**
MEA	3.48	4.28	-0.80	-5.65	< 0.001**
LMA	1.93	2.07	-0.14	-1.22	0.22

14-15 years old					
N	542	497	-	-	-
AMAS	7.06	8.02	-0.96	-2.81	0.005**
MEA	4.10	4.87	-0.77	-4.06	<0.001**
LMA	2.95	3.15	-0.20	-1.07	0.28

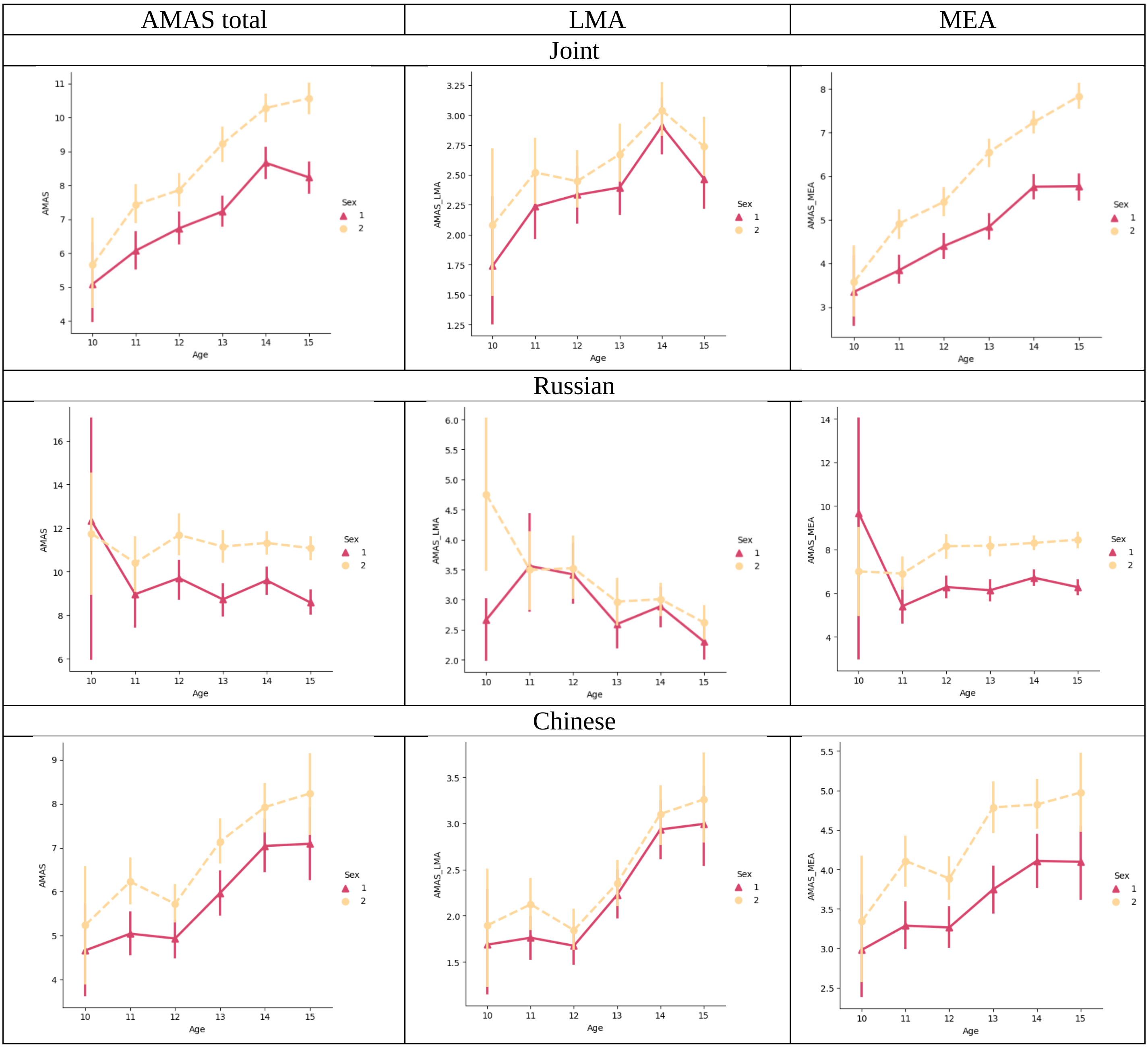

Figure 8. Interactions between age and gender for AMAS, LMA, MEA in the joint, Russian and Chinese samples
